# α-Synuclein Suppression by Targeted Small Interfering RNA in the Primate Substantia Nigra

**DOI:** 10.1371/journal.pone.0012122

**Published:** 2010-08-11

**Authors:** Alison L. McCormack, Sally K. Mak, Jaimie M. Henderson, David Bumcrot, Matthew J. Farrer, Donato A. Di Monte

**Affiliations:** 1 German Center for Neurodegenerative Diseases (DZNE), Bonn, Germany; 2 SRI International, Menlo Park, California, United States of America; 3 The Parkinson's Institute, Sunnyvale, California, United States of America; 4 Stanford University, Stanford, California, United States of America; 5 Alnylam Pharmaceuticals, Cambridge, Massachusetts, United States of America; 6 Mayo Clinic College of Medicine, Jacksonville, Florida, United States of America; Mayo Clinic, United States of America

## Abstract

The protein α-synuclein is involved in the pathogenesis of Parkinson's disease and other neurodegenerative disorders. Its toxic potential appears to be enhanced by increased protein expression, providing a compelling rationale for therapeutic strategies aimed at reducing neuronal α-synuclein burden. Here, feasibility and safety of α-synuclein suppression were evaluated by treating monkeys with small interfering RNA (siRNA) directed against α-synuclein. The siRNA molecule was chemically modified to prevent degradation by exo- and endonucleases and directly infused into the left substantia nigra. Results compared levels of α-synuclein mRNA and protein in the infused (left) *vs.* untreated (right) hemisphere and revealed a significant 40–50% suppression of α-synuclein expression. These findings could not be attributable to non-specific effects of siRNA infusion since treatment of a separate set of animals with luciferase-targeting siRNA produced no changes in α-synuclein. Infusion with α-synuclein siRNA, while lowering α-synuclein expression, had no overt adverse consequences. In particular, it did not cause tissue inflammation and did not change (i) the number and phenotype of nigral dopaminergic neurons, and (ii) the concentrations of striatal dopamine and its metabolites. The data represent the first evidence of successful anti-α-synuclein intervention in the primate substantia nigra and support further development of RNA interference-based therapeutics.

## Introduction

Several lines of evidence link the protein α-synuclein to human neurodegenerative diseases. Point mutations in the α-synuclein gene (*SNCA*) and increased expression of wild-type α-synuclein due to *SNCA* multiplication mutations are causally associated with familial forms of parkinsonism and dementia [1]–[5]. α-Synuclein is also implicated in the pathogenesis of non-familial diseases such as idiopathic Parkinson's disease (PD), dementia with Lewy bodies (DLB) and multiple system atrophy (MSA). In PD and DLB, α-synuclein is a major component of the intraneuronal inclusions called Lewy bodies and Lewy neurites that accumulate extensively throughout the brain and have been suggested to underlie disease development and progression [6], [7]. A widespread diffusion of oligodendroglial cytoplasmic inclusions composed of filamentous α-synuclein is observed in MSA and has recently been proposed as a criterion for definite post-mortem disease diagnosis [8], [9]. The tendency of α-synuclein to aggregate, which is well documented by both *in vitro* and *in vivo* experimental work, likely explains the formation of these neuronal and glial pathologic inclusions [10]–[12]. It may also result in a gain of toxic function of the protein and therefore contribute to neuronal injury and degeneration [10]–[13].

The toxic properties of α-synuclein together with its involvement in pathogenetic processes point to this protein as a promising target for new therapeutic intervention against PD and other α-synuclein-related neurodegenerative disorders (collectively referred to as α-synucleinopathies) [14]. Because clinical and experimental findings indicate that higher levels of α-synuclein promote its toxic potential [3], [11]–[13], it is also reasonable to postulate that neuroprotective effects could be achieved by suppressing neuronal expression of the protein. RNA interference (RNAi) is a pathway of post-transcriptional gene silencing that, since its characterization in the late 1990s, has given researchers the opportunity to study the consequences of selective gene knockdown experimentally. As importantly, exploitation of this pathway has been proposed for the treatment of diseases that feature the expression of specific harmful proteins; based on the previous considerations, it is not surprising that α-synucleinopathies are often listed among these diseases [15].

Earlier investigations have tested RNA molecules targeting α-synuclein *via* RNAi in neuron-like cell cultures as well as in rodent models *in vivo* [16]–[19]. In the latter, short hairpin (sh) α-synuclein RNA delivered *via* a lentiviral vector was found to silence ectopic expression of human α-synuclein in the rat striatum, and small interfering RNA (siRNA) directed against α-synuclein reduced the expression of endogenous α-synuclein after a two-week infusion into the mouse hippocampus [17], [18]. No signs of toxicity were reported as a consequence of these treatments. More recently, two α-synuclein siRNAs embedded as shRNAs in adeno-associated virus vectors were unilaterally injected into the rat substantia nigra pars compacta [19]. Quite surprisingly, this administration was found to cause a significant loss of nigrostriatal dopaminergic neurons. In the present study, the effects of siRNA directed against α-synuclein were tested in the monkey substantia nigra. Several lines of consideration underscore the relevance of this work. To the best of our knowledge, no study to date has assessed the feasibility, effectiveness and safety of RNAi-based suppression of α-synuclein in the primate brain. Moreover, the apparent inconsistency of rodent data warrants further evaluation in an animal model highly pertinent to humans. Finally, investigations using monkey models are justified by α-synuclein features unique to the primate brain (e.g. age-related upregulation) that may contribute to pathogenetic processes [20]–[22]. siRNA directed against α-synuclein was continuously infused into the substantia nigra of squirrel monkeys over a one-month period. Post-mortem evaluation determined if this treatment decreased neuronal expression of both α-synuclein mRNA and protein, and if it was associated with inflammatory tissue reaction. The number of nigral dopaminergic neurons and the concentration of striatal dopamine were also assayed to rule out any adverse effect of α-synuclein siRNA on dopaminergic cell function/integrity.

## Methods

### Ethics Statement

Monkeys were housed at The Parkinson's Institute in a facility constructed according to AAALAC guidelines. Animal welfare and experimental protocols were in accordance with the guidelines of the National Institutes of Health and the Office of the Prevention of Research Risks and were approved by the Institutional Animal Care and Use Committee at The Parkinson's Institute. Monkeys had free access to water and a daily diet of monkey chow and fresh fruit. They were evaluated weekly for general health (e.g. weight) and, if necessary, treated by an attending Veterinarian. During the surgical procedure, monkeys were anesthetized with ketamine and isoflurane. To prevent post-surgery pain/discomfort, animals were treated with an analgesic drug for at least 24 hours. After surgery, they were also assessed daily for weight, food consumption, general health and appearance, and urine and fecal output. Monitoring of these parameters revealed no significant post-surgery side effects. The animals were euthanized using procedures consistent with the recommendations of the Panel of Euthanasia of the American Veterinary Medical Association.

### Sequencing

Total RNA was isolated from cerebellar tissue from 20 squirrel monkeys, and cDNA prepared using SuperScript III First Strand Synthesis kit (Invitrogen, Carlsbad, CA). PCR primers were designed (forward 5′-CGACGACAGTGTGGTGTAAA-3′ and reverse 5′-GCACATTGGAACTGAGCACT-3′) to amplify the entire *SNCA* gene. Standard PCR reactions were performed using Qiagen PCR reagents (Qiagen, Valencia, CA) and a 60-50°C touchdown PCR protocol. Reactions contained a final concentration of 0.8 µM of each primer, 1 unit of Taq polymerase and 5 µl of Q solution (Qiagen). PCR products were purified using AMPure (Agencourt Biosciences, Beverly, MA) and then sequenced in both directions using the Big Dye Terminator v3.1 Cycle Sequencing kit (Applied Biosystems, Carlsbad, CA). Sequencing reactions were purified using CleanSEQ (Agencourt Biosciences) and analyzed on an ABI3730 Genetic Analyzer (Applied Biosystems).

### Animal Procedures and Tissue Preparation

A total of 6 feral adult squirrel monkeys (*Saimiri scuireus*) of both sexes (4 females and 2 males) were purchased from Worldwide Primates (Miami, FL) and underwent stereotactic surgery. A cannula (20 mm length, 0.18 mm internal diameter and 0.36 mm outer diameter, Plastics One, Roanoke, VA) was implanted (18.7 mm deep) into the left midbrain at the following co-ordinates: +4.75 mm anterior and +2.0 mm lateral to bregma [23]. An Alzet osmotic mini-pump (2004 model, Durect Inc., Cupertino, CA) containing siRNA solution (27 mg/ml in 0.1 M phosphate buffered saline, pH 7.4) was attached to the cannula *via* catheter tubing and placed under the skin in the mid-scapular region. Pumps delivered the siRNA into the left substantia nigra for a 4-week period at a flow rate of 5.4 µg/hour.

Four weeks after cannula implantation, the animals were euthanized, and their brains were rapidly removed and dissected. A tissue block encompassing the entire substantia nigra from each hemisphere was prepared and fixed in 4% paraformaldehyde in phosphate buffered saline, cryoprotected and frozen. Each block was cryostat-cut into 40 µm-thick sections. The two cerebral hemispheres were cut into 2 mm-thick serial sections using a brain mold and snap-frozen.

### *In situ* Hybridization

α-Synuclein RNA probes labeled with digoxigenin (dig-11-UTP, Roche Applied BioSciences, Indianapolis, IN) were prepared by *in vitro* transcription using T7 RNA polymerases (Ambion, Austin, TX) [24]. Midbrain sections from both hemispheres were processed in parallel and incubated overnight at 60°C with either antisense or sense probes. Hybridization signal was detected using an anti-digoxigenin antibody, 5-bromo-4-chloroindolyl-phosphatase and nitroblue tetrazolium (Roche Applied Biosciences).

### Quantitative PCR

The substantia nigra was dissected from 40 µm cryostat-cut midbrain sections. Total RNA was extracted using RNeasy FFPE kit (Qiagen) and reverse-transcribed in the presence of a random hexamer primer into cDNA (SuperScript III first-strand synthesis for reverse transcription PCR kit, Invitrogen). The resulting cDNA sample was diluted and used as template DNA for quantitative PCR (qPCR) reactions (PRISM 7000 sequence detection system, Applied Biosystems). Data were normalized to β-actin content and expressed as fold change over a control sample from the right (untreated) substantia nigra. Two gene-specific primer sequences were designed with Primer Express software (Applied Biosystems) based on the human α-synuclein sequence (GenBank: BC013293.2). To ensure reliability of the results, analyses were performed using two separate sets of primers. The first set was: 5′-AAGGACCAGTTGGGCAAGAATG-3′ and 5′-TGCCTGTGGATCCTGACAATGA-3′. The second set was: 5′- AAAGGACCAGTTGGGCAAG -3′ and 5′-TCCAGAATTCCTTCCTGTGG-3′. For β-actin, the primers used were 5′-CAGCAGATGTGGATCAGCAAG-3′ and 5′-GCATTTGCGGTGGACGAT-3′ [25]. The absence of unwanted by-products was confirmed by automated melting-curve analysis and agarose gel electrophoresis.

### Stereology and Immunohistochemistry

All quantitative analyses were performed by an investigator blinded to the animal treatment. Stereological cell counting was carried out as previously described [26]. Briefly, every twelfth section throughout the substantia nigra (8–9 sections per hemisphere) was immunostained with an anti-tyrosine hydroxylase (TH) antibody (1∶600; Pel Freez, Rogers, AK) using the avidin-biotin immunoperoxidase method (Vector, Burlingame, CA) with 3,3′-diaminobenzidine (DAB) as the chromagen. Sections were counterstained with cresyl violet and the number of dopaminergic neurons counted using the optical fractionator technique (StereoInvestigator, MBF Bioscience, Williston, VT). The substantia nigra was delineated at low magnification on each section. It was then systematically sampled with a 100× oil immersion lens using the nucleolus as the sampling unit. Dopaminergic neurons were identified by the presence of TH immunoreactivity and/or neuromelanin pigmentation [26]. The coefficient of error ranged between 0.08 and 0.09.

Two midbrain sections per hemisphere were used for densitometric quantification of nigral α-synuclein immunoreactivity. Sections were immersed in 5% normal serum prior to incubation in mouse-anti-α-synuclein (1∶6,000; LabVision, Fremont, CA). Immunostaining was detected using the avidin-biotin immunoperoxidase method with DAB as the chromagen. The right and left substantia nigra were delineated at low magnification and then systematically sampled using a 100× oil immersion lens. A grid size of 380×810 um^2^ was used to generate approximately 15 evenly spaced nigral sampling areas per hemisphere. Digital images of these areas were acquired using a camera system attached to an Olympus BX51 microscope. They were then analyzed with SimplePCI software (Compix Inc., Lake Oswego, OR) to determine the optical density of α-synuclein immunoreactivity. All parameters, such as light intensity, were standardized and kept consistent throughout the analysis. For each animal, optical densities of the sampling areas from the right or left hemisphere were averaged.

For visualization of microglia, midbrain sections were immunostained with an antibody recognizing the microglial marker ionizing calcium-binding adaptor molecule 1 (Iba-1) (1∶1,000; Biocare Medical, Concord, CA) and counterstained with cresyl violet. To assess whether cannula implantation and/or siRNA infusion caused microglial changes, the number of Iba-1-positive cells with morphological features of resting or activated microglia was counted in the substantia nigra. For each animal, counts were performed on two sections collected at the site of the exit of the cannula in the left hemisphere or at the corresponding anatomical level on the right side. Values from the two sections were then averaged.

### Neurochemical Measurements

For each monkey, four tissue punches (approximately 5 mg each) were collected from the caudate (lateral and medial) and putamen (ventral and dorsal) at the level of the anterior commissure. After immediate sonication in 0.4 N perchloric acid, sample homogenates were centrifuged at 15,000×g for 12 min at 4°C. Protein levels were assayed in the pellets, while supernatants were used for measurements of dopamine, dihydroxyphenylacetic acid (DOPAC) and homovanillic acid (HVA) by HPLC coupled to electrochemical detection [26].

### Statistical Analysis

Data are presented as mean ± SEM. Paired Student's *t*-test was performed to compare values obtained from the right (untreated) *vs.* left (siRNA-infused) substantia nigra. P<0.05 was considered a significant difference.

## Results

### Effect of siRNA Infusion on Nigral α-Synuclein Expression

**Figure 1:**
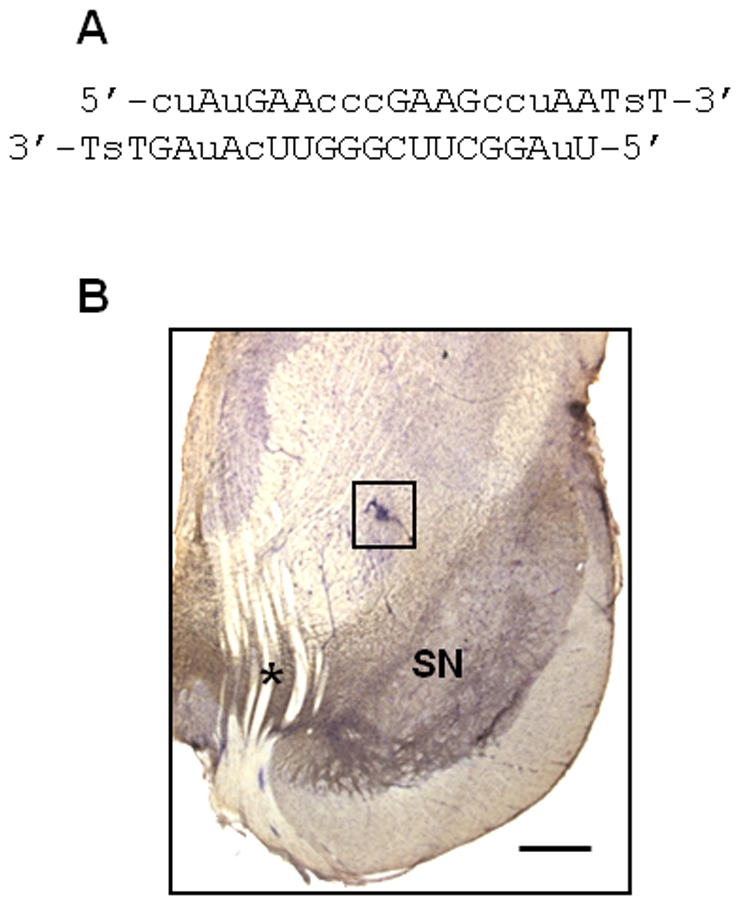
Treatment of squirrel monkeys with siRNA. Animals were unilaterally implanted with a cannula connected to an Alzet minipump delivering siRNA into the left substantia nigra. (**A**) Sequence of the α-synuclein siRNA. “A,C,G,U” indicate ribonucleotides, “T” designates deoxythymidine, “c” and “u” specify 2′-O-Me-modified pyrimidines and “s” denotes a phosphorothioate linkage. (**B**) Midbrain sections were immunostained for tyrosine hydroxylase (brown) and counterstained with cresyl violet (purple). A representative section shows placement of the cannula approximately 1 mm dorsal to the substantia nigra (SN). The location of the cannula is indicated by the square box, and the asterisk denotes the exit of the third nerve. Scale bar = 800 µm.

DNA was extracted from cerebellar samples of squirrel monkeys and used to deduce the nucleotide sequence of the α-synuclein gene (*SNCA*). A 21-base pair siRNA duplex was designed and synthesized to specifically target the squirrel monkey *SNCA* transcript; oligonucleotides were chemically modified to prevent degradation by exo- and endonucleases (Fig. 1A). Three monkeys (2 females and one male) were treated with this α-synuclein siRNA. The siRNA was infused unilaterally for a period of 4 weeks *via* a cannula implanted approximately 1 mm dorsal to the left substantia nigra and connected to an Alzet mini-pump (Fig. 1B). To rule out the possibility of non-specific effects of siRNA infusion, a separate group of 3 monkeys (2 females and one male) received a 4-week nigral infusion with control siRNA targeting a futile protein, i.e. firefly luciferase.

**Figure 2:**
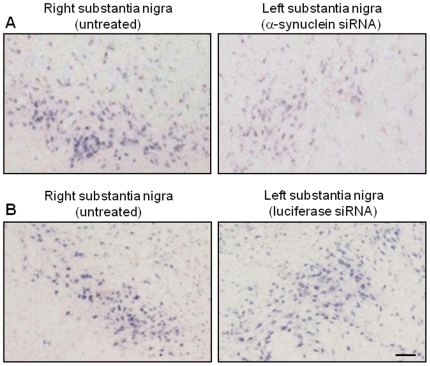
Reduction of α-synuclein mRNA in the substantia nigra infused with α-synuclein siRNA. Squirrel monkeys received a unilateral nigral infusion of siRNA targeting α-synuclein (**A**) or luciferase (**B**). Midbrain sections at the level of the exit of the 3^rd^ nerve were used for α-synuclein *in situ* hybridization using digoxigenin-labeled antisense riboprobes. Representative images compare α-synuclein mRNA in the right (untreated) *vs.* left (siRNA-infused) substantia nigra. Scale bar = 100 µm.

**Figure 3:**
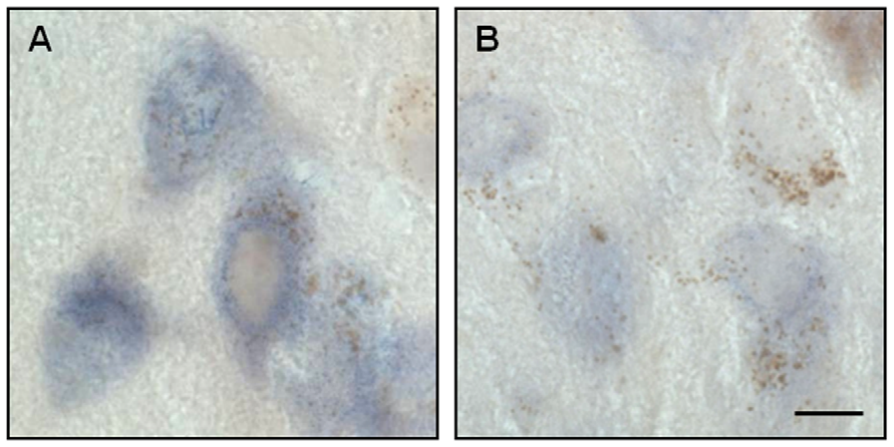
α-Synuclein siRNA decreases α-synuclein mRNA within pigmented nigral neurons. siRNA against α-synuclein was infused into the left substantia nigra of squirrel monkeys. Right (**A**) and left (**B**) midbrain sections at the level of the exit of the 3^rd^ nerve were used for α-synuclein *in situ* hybridization. Representative images show nigral dopaminergic neurons containing neuromelanin (brown granules). The hybridization signal (purple) was markedly reduced in the left (siRNA-infused) as compared to the right (untreated) hemisphere. Scale bar = 10 µm.

Post-mortem analysis assessed the effect of siRNA administration on α-synuclein mRNA by both *in situ* hybridization and qPCR. α-Synuclein *in situ* hybridization was performed on a set of midbrain sections at the level of the exit of the 3^rd^ nerve encompassing the mid substantia nigra. Sections from the left (siRNA-infused) and right (untreated) hemispheres, which were processed in parallel, showed an overt difference in mRNA labeling, with less robust signal intensity and decreased number of labeled neurons in the left substantia nigra of all 3 animals receiving α-synuclein siRNA (Fig. 2A). In contrast, no apparent change in hybridization signal was detected between the left and right side of the brain in the 3 monkeys infused with luciferase siRNA (Fig. 2B). Nigral dopaminergic neurons in the primate brain can be identified due to their content of neuromelanin pigment [26]. Higher magnification images of right midbrain sections labeled for α-synuclein mRNA showed a robust signal within neuromelanin-loaded cells (Fig. 3A). The intensity of this signal was markedly reduced after infusion with α-synuclein siRNA in the left hemisphere (Fig. 3B), consistent with the interpretation that suppression of α-synuclein mRNA occurred within the cell body of nigral dopaminergic neurons.

**Figure 4:**
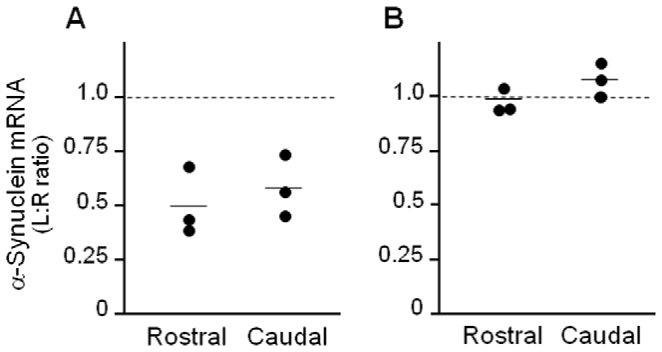
Measurement of nigral α-synuclein mRNA by qPCR. Squirrel monkeys received unilateral nigral infusion of siRNA targeting α-synuclein (**A**) or luciferase (**B**). Nigral tissue was dissected from midbrain sections rostral and caudal to the exit of the 3^rd^ nerve. Values are the ratio of α-synuclein mRNA levels measured by qPCR in the left (siRNA-infused) and right (untreated) substantia nigra (L∶R ratio). Bars represent mean values.

Quantitative PCR was used to measure changes in α-synuclein mRNA caused by siRNA administration. For each monkey, samples of the left (siRNA-infused) and right (untreated) substantia nigra were dissected from two midbrain sections, one anterior and one posterior to the third nerve. RNA was extracted from these samples, and analyses compared the effects of siRNA infusion between the two hemispheres as well as in the rostral and caudal portions of the substantia nigra. Treatment with α-synuclein but not luciferase siRNA caused a significant reduction of α-synuclein expression, as reflected by a decrease in the left∶right mRNA ratio (paired Student's *t* test, *P* = 0.03; Fig. 4). Within the left hemisphere, the extent of this effect was 50% in the anterior and 42% in the posterior substantia nigra (not statistically different; Fig. 4A).

**Figure 5:**
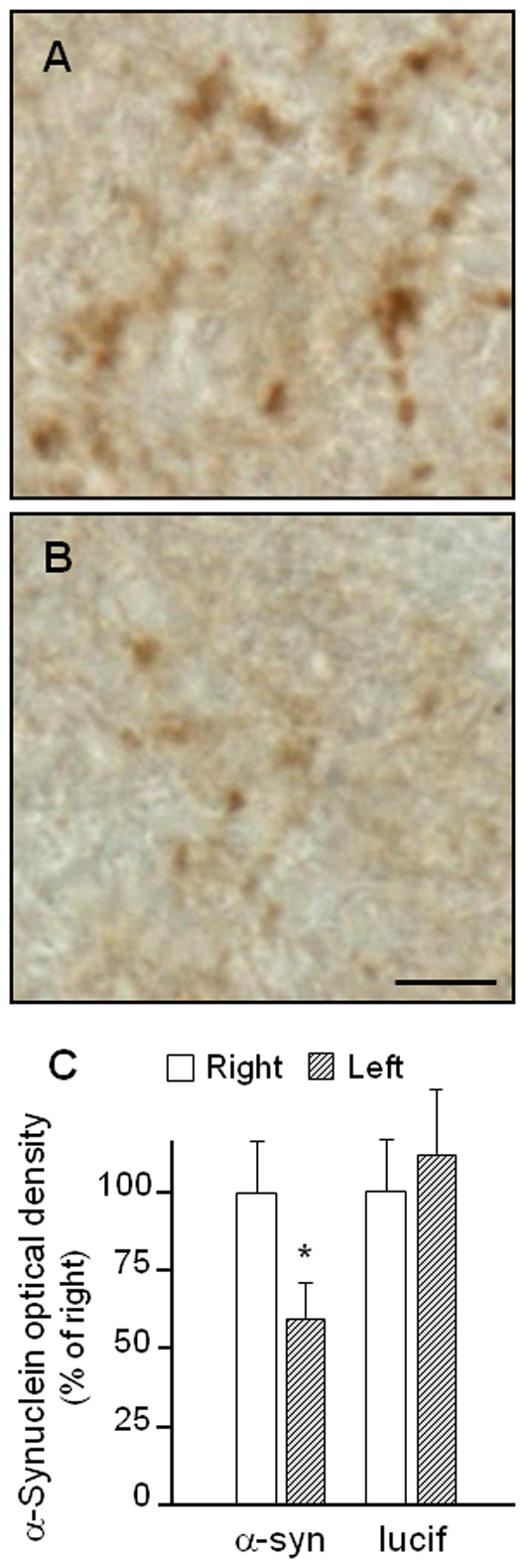
Effect of α-synuclein siRNA on α-synuclein protein in the monkey substantia nigra. α-Synuclein or luciferase siRNA was unilaterally infused into the left substantia nigra. Midbrain sections were immunostained with an antibody against α-synuclein. Representative images from an animal receiving α-synuclein siRNA show more robust α-synuclein immunoreactivity within the neuropil of the right (untreated, **A**) *vs.* left (siRNA-infused, **B**) substantia nigra. Scale bar = 5 µm. (**C**) Optical density measurements of nigral α-synuclein immunoreactivity. Data are expressed as percent of the control value in the right (untreated) substantia nigra and represent mean ± SEM. A significant decrease is caused by α-synuclein but not luciferase siRNA in the left (siRNA-infused) hemisphere. *p<0.03.

siRNA-induced changes in α-synuclein protein were assessed by immunohistochemistry. Features of α-synuclein distribution have been previously characterized in the monkey substantia nigra: α-synuclein immunoreactivity labels the neuropil in a punctate pattern, with little or no staining of neuronal cell bodies [22]. We confirmed this pattern and showed marked α-synuclein immunoreactivity in midbrain sections from the right hemisphere (Fig. 5A). This immunoreactivity was decreased in all 3 monkeys infused with α-synuclein siRNA (Fig. 5B); indeed, densitometric analysis revealed a significant 40% difference between the siRNA-infused (left) and untreated (right) substantia nigra (Fig. 5C). In contrast, no change in α-synuclein staining was observed as a consequence of luciferase siRNA administration (data not shown).

### Lack of Adverse Effects of α-Synuclein siRNA Infusion

**Figure 6:**
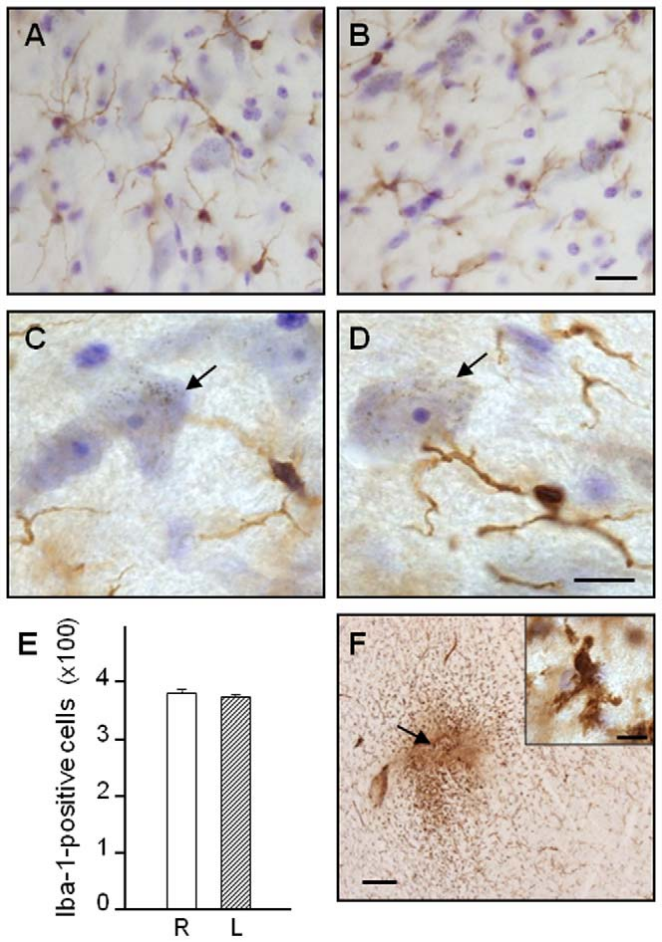
Lack of microglial activation following siRNA infusion. α-Synuclein siRNA was unilaterally infused through a cannula positioned approximately 1 mm dorsal to the substantia nigra. Representative midbrain sections were immunostained for microglial cells using an antibody against ionizing calcium-binding adaptor molecule 1 (Iba-1, brown) and counterstained with cresyl violet (purple). Images are from the right (untreated, **A** and **C**) and left (siRNA-infused, **B** and **D**) substantia nigra. At higher magnification (**C** and **D**), Iba-1-positive cells with morphological features of resting microglia are shown close to dopaminergic neurons containing neuromelanin (black granules). The arrows indicate one of these neurons in each panel. Scale bars = 20 µm (A and B) and 10 µm (C and D). (**E**) The number of Iba-1-immunoreactive cells was counted in the right (R) and left (L) substantia nigra. Data are shown as mean ± SEM. (**F**) A representative section from the left midbrain shows Iba-1 immunoreactivity close to the tip of the infusion cannula (arrow) but not within the nearby parenchyma. This robust immunoreactivity was observed within cells with morphological characteristics of activated microglia (inset). Scale bars = 250 µm (panel F) and 10 µm (inset).

A variety of markers of systemic toxicity (e.g., animal's weight and temperature) were monitored throughout the course of the experiments. No complications or adverse effects were observed during surgical procedure, post-operative recovery/treatment and time of infusion. Post-mortem analysis of midbrain sections revealed that α-synuclein siRNA infusion did not cause overt tissue inflammation in the form of microglial activation. After staining with Iba-1, resting and activated microglial cells were identified and counted based on their morphological characteristics. Within the substantia nigra, the vast majority of Iba-1-immunoreactive cells featured small bodies and thin ramifications reminiscent of resting microglia; their number was unchanged between the two hemispheres (Fig. 6A–E). Microglial cells with amoeboid-shaped cytoplasm and thick, short ramifications were present around the cannula tract (Fig. 6F). These activated cells were rarely observed, however, within the nearby parenchyma as well as in sections throughout the left (siRNA-infused) and right (untreated) midbrain.

**Figure 7:**
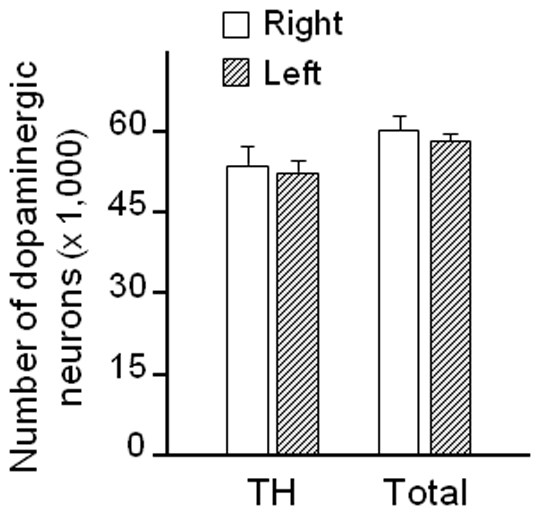
The number of nigral dopaminergic neurons is not affected by siRNA-induced α-synuclein suppression. Squirrel monkeys received a unilateral nigral infusion of siRNA targeting α-synuclein. Both the number of TH-immunoreactive cells and the total number of dopaminergic neurons were counted stereologically in the substantia nigra. Values (mean ± SEM) were not different between the right (untreated) and left (siRNA-infused) hemisphere.

To rule out neuronal damage as a consequence siRNA infusion, the number of dopaminergic cells was counted in the right and left substantia nigra of monkeys receiving unilateral infusion of α-synuclein siRNA. The number of TH-immunoreactive neurons, which was estimated using stereological methods, was found to be approximately 53,000 and not significantly different between the two hemispheres (Fig. 7). Data from earlier reports indicate that TH-based counting does not identify all dopaminergic neurons in the monkey substantia nigra; a subpopulation of these cells is devoid of TH immunoreactivity but can still be recognized by their neuromelanin load [26]. We therefore counted these additional cells and calculated the total number of dopaminergic neurons by adding the number of TH-positive cells plus the number of neuromelanin-containing (TH-negative) neurons. Consistent with previous results [24], [26], this total count was approximately 60,000 in the right and left hemispheres, indicating that infusion with α-synuclein siRNA affected neither the number nor the phenotype (i.e. TH-positive *vs.* TH-negative) of nigral neurons (Fig. 7).

A final set of measurements was carried out in samples from the monkey caudate and putamen to determine if α-synuclein suppression changed the concentrations of striatal dopamine and its metabolites DOPAC and HVA. Unilateral administration of α-synuclein siRNA did not cause any significant neurochemical alteration; dopamine, DOPAC and HVA levels were similar in the right and left striatum (Table 1).

**Table 1:** Levels of striatal dopamine, DOPAC and HVA are unaffected by α-synuclein siRNA infusion.

Region	Dopamine		DOPAC		HVA	
	Right	Left	Right	Left	Right	Left
**LC**	142±9	155±5	11.6±1.6	11.7±0.8	61.1±5.9	63.8±6.4
**MC**	158±8	167±12	11.6±1.2	11.2±1.8	57.8±5.1	52.1±7.7
**DP**	174±7	192±10	9.8±1.0	9.8±0.9	83.3±8.9	93.7±6.8
**VP**	158±7	167±9	13.2±2.4	14.0±1.2	82.4±15	90.6±14

α-Synuclein siRNA was unilaterally infused into the left substantia nigra. Values are expressed as ng/mg protein. Data are shown as mean ± SEM. LC, lateral caudate; MC, medial caudate; DP, dorsal putamen; VP, ventral putamen.

## Discussion

Results of this study provide the first documentation that α-synuclein expression can be significantly reduced *via* RNAi in the primate brain. siRNA targeting α-synuclein suppressed both α-synuclein mRNA and protein. The selectivity of this effect was demonstrated by experiments in which monkeys were treated with luciferase rather than α-synuclein siRNA. Lack of changes in the brain of these control animals underscores that α-synuclein silencing requires administration of *SNCA*-complementary siRNA. Data also reveal that siRNA directed against α-synuclein is capable of decreasing its expression within neuronal cells highly susceptible to neurodegenerative processes, i.e. nigrostriatal dopaminergic neurons. siRNA-induced reduction of α-synuclein mRNA was observed in midbrain sections encompassing the rostral, mid- and caudal substantia nigra. This finding indicates that the infusion protocol of naked siRNA employed in this study was effective in lowering α-synuclein throughout the monkey substantia nigra.

Our present data show a 40–50% reduction of nigral α-synuclein. Based on *in vitro* and *in vivo* evidence [16], [19], more pronounced α-synuclein suppression could conceivably be achieved by increasing the dosage of siRNA, testing other siRNA molecules or using a different method for siRNA delivery (e.g. viral vector). Further studies should confirm this concept in the primate substantia nigra. Additional work is also warranted to confirm and extend data in rodents indicating that α-synuclein silencing is sustained even after cessation of siRNA treatment; when α-synuclein siRNA was infused into the rat hippocampus, levels of α-synuclein mRNA returned to normal levels by three weeks post-infusion [18].

Although more powerful siRNA molecules could be evaluated in our animal model to achieve a greater reduction of neuronal α-synuclein, several lines of argument underscore the relevance of the present results. α-Synuclein is a relatively abundant protein in the CNS where it is thought to play a role in synaptic neurotransmission and plasticity and has been found to regulate pathways involved in dopaminergic activity [27]–[29]. The dual behavior of α-synuclein, involving both toxic and physiological properties, has important implications for the development of anti-α-synuclein therapeutics. Less-than-complete α-synuclein suppression, as achieved in our study, would be less likely to have a negative impact on the normal function of the protein. This partial effect could still be sufficient, however, to counteract α-synuclein's toxic potential and to avert conditions that have been implicated in α-synuclein pathology. Deleterious properties such as the tendency of α-synuclein to aggregate are concentration-dependent and would be inhibited at lower protein expression [11]. Moreover, as a consequence of reduced α-synuclein burden, neuronal mechanisms of protein homeostasis, such as proteasomal and lysosomal degradation pathways [30]–[32], could more effectively prevent α-synuclein-dependent pathology.

Another important corollary to the concept of α-synuclein playing both a physiological and pathological role is that anti-α-synuclein approaches need to be evaluated for possible adverse consequences. Results of our present study are consistent with the interpretation that siRNA-induced α-synuclein reduction is devoid of overt side effects. Monkeys infused with α-synuclein siRNA showed no signs of either systemic or tissue-specific toxicity. The latter, as assessed in the form of lack of microglial activation, is particularly relevant given that (i) nigral tissue is highly vulnerable to inflammatory-mediated injury, and (ii) neuroinflammation is thought to contribute to pathogenetic process in PD and other neurodegenerative diseases [33], [34]. Thus, one of the potential benefits of treatment with naked siRNA as compared, for example, to viral vector-mediated delivery of siRNA is a lack of tissue reaction to the directly infused molecule.

Further supporting the safety of this treatment, the total number of dopaminergic neurons was found to be unchanged in the substantia nigra infused with α-synuclein siRNA. The count of TH-immunoreactive cells was also unaffected, suggesting that α-synuclein inhibition did not cause any marked loss of nigral TH expression. Quite noteworthy are the results of measurements of dopamine and its metabolites as markers of dopaminergic function/integrity at the level of striatal terminals. In an earlier investigation, alterations in electrically evoked dopamine release were found using striatal slice preparations from mice lacking α-synuclein [28]. These mutant animals were also characterized by a significant decrease in striatal dopamine, suggesting a role of α-synuclein in presynaptic regulation of dopaminergic neurotransmission [28], [35]. Dopamine concentrations were measured in our study in the monkey caudate and putamen and values were compared in the siRNA-infused (left) *vs.* untreated (right) side of the brain. Results showed that α-synuclein suppression did not affect striatal dopamine since levels of this neurotransmitter as well as levels of its metabolites DOPAC and HVA were not different in the left and right striatum. Several explanations could underlie the apparent inconsistency between previous findings and the current data, including the fact that experiments involved different animal species (rodent *vs.* primates). Most likely, however, changes in striatal dopamine may be dependent upon the extent and/or duration of α-synuclein deficiency; no effect was observed after partial and temporary α-synuclein reduction in our siRNA-treated monkeys, whereas a dopamine loss resulted from lifelong ablation of α-synuclein in mutant mice.

Similar considerations could apply to the evaluation of data recently published indicating that viral vector-based RNAi targeting α-synuclein in the rat substantia nigra caused dopaminergic cell loss and behavioral changes consistent with nigrostriatal impairment [19]. These dramatic effects, which contrast with the lack of overt toxicity in the present study, may further emphasize the importance of partial *vs.* more pronounced silencing of α-synuclein. Indeed, nigrostriatal damage was observed in rats in which siRNA treatment resulted in >90% reduction of α-synuclein mRNA [19]. Other important differences between the earlier report and our present investigation concern the animal species in which nigral α-synuclein was silenced and the method of siRNA delivery, i.e. injection of an adeno-associated virus vector *vs.* infusion of naked siRNA.

Taken together, the results of this study provide important new evidence that RNAi-mediated suppression of α-synuclein in the primate brain is feasible and relatively safe and should therefore be further pursued for therapeutic purposes against human α-synucleinopathies. Potential caveats include the extent and duration of α-synuclein silencing. Would partial suppression be sufficient to achieve neuroprotection and, if not, could a close-to-complete reduction of α-synuclein pose safety risks? In the present study, siRNA infusion was maintained over a period of one month. However, treatment of chronic α-synucleinopathies will likely involve long-term anti-α-synuclein intervention. Thus, detailed evaluation of the consequences of protracted α-synuclein suppression is needed to confirm sustained siRNA efficacy and to rule out the possibility that adverse side effects may become evident only after prolonged silencing of α-synuclein expression.
